# Breast Cancer Screening Among Adult Women in China, 2010

**DOI:** 10.5888/pcd10.130136

**Published:** 2013-11-07

**Authors:** Baohua Wang, Minfu He, Limin Wang, Michael M. Engelgau, Wenhua Zhao, Linhong Wang

**Affiliations:** Author Affiliations: Baohua Wang, Minfu He, Limin Wang, Wenhua Zhao, Chinese Center for Disease Control and Prevention, Beijing, China; Michael M. Engelgau, Centers for Disease Control and Prevention, Atlanta, Georgia.

## Abstract

**Introduction:**

Breast cancer is the most frequently diagnosed type of cancer among women in China. However, China does not have a national screening program or national screening guidelines. Little information on participation in breast cancer screening among Chinese women is available.

**Methods:**

We used data from the 2010 China Chronic Disease and Risk Factor Surveillance System that included 53,513 women aged 18 years or older. Women were asked about breast cancer screening participation (any type of screening method), and we examined screening participation rates. We adjusted estimates and performed multivariable logistic regression to adjust for potential confounders.

**Results:**

Overall, 21.7% (95% confidence interval [CI], 19.2%–24.2%) of respondents reported previous breast cancer screening. The participation rates were highest among women aged 30 to 39 years (30.7%; 95% CI, 26.9%–34.4%) and lowest among women 70 years or older (6.3%; 95% CI, 5.1%–7.6%). Compared with women living in the western region, women in the eastern region were 1.5 times more likely to be screened (adjusted odds ratio [OR], 1.5; 95% CI, 1.2–2.0). Compared with women without insurance, women with urban insurance were more likely to be screened (prevalence ratio = 2.6; 95% CI, 2.3–3.0) and be screened within the last 2 years (OR = 1.3; 95% CI, 1.0–1.7; *P* = .04).

**Conclusion:**

Breast cancer screening participation rates among Chinese women were low and varied greatly by age, region, and insurance status. Comprehensive and prioritized strategies are needed to improve breast cancer screening participation among older women, those without medical insurance, and those living in the west.

## Introduction

Among women in China, breast cancer is the most common and the leading cause of death from all cancers. Mortality from breast cancer has been rising in recent years. Data from the Second and Third National Retrospective Sampling Survey of Death Causes in China estimated that breast cancer–related mortality increased between 1990–1992 and 2004–2005 from 3.84 per 100,000 to 5.09 per 100,000 population (1). In 2008, approximately 169,000 new cases of breast cancer were diagnosed, and it was the most frequently diagnosed type of cancer among Chinese women. During that same year, breast cancer–related mortality was 5.7 per 100,000 population, resulting in approximately 44,900 deaths (2).

The World Health Organization (WHO) recommends cancer screening only when resources are available to reach a high proportion of the at-risk population and to cover the costs of diagnosis and management of the abnormalities detected (3). Therefore, breast cancer screening using mammography is recommended only among women aged 50 to 69 years in high-resource settings (4). Other less resource-dependent measurements have been proposed in other settings for early detection of breast cancer, such as clinical breast examination, combined with other strategies, to improve diagnosis and treatment of the disease (5).

Breast cancer screening programs (screening methods include mammography, ultrasound, or clinical breast examination) are available in most provinces of China, although coverage is highly variable. Currently in China, however, neither an organized screening program operating nationwide nor national breast cancer screening guidelines exist (6). To align ongoing national efforts, better understanding of the status of breast cancer screening across China is needed. This understanding will facilitate development of comprehensive breast cancer prevention and control strategies. In this study, we aimed to describe breast cancer screening participation among women in China and examine key factors associated with screening.

## Methods

### Study subjects

We obtained data from the 2010 China Chronic Disease and Risk Factor Surveillance System (CCDRFSS). This surveillance system monitors chronic diseases, health behaviors, access to health care, and preventive health care. A face-to-face interview is conducted every 3 years using a nationally representative random sampling design based on the China National Disease Surveillance Points (DSP) system. Detailed descriptions of the DSP system are published elsewhere (7). The 2010 CCDRFSS covered all 31 provinces, autonomous regions, and municipalities, and included 162 surveillance points (63 districts from urban areas and 99 districts from rural areas). A multistage, stratified-cluster, random sampling method was used. In the first stage of sampling, 4 townships were randomly selected from each surveillance point using the method of probability proportional to size (PPS). In the second stage, 3 villages or communities were sampled from each selected township by the PPS method. In the final sampling stage, a residential group (at least 50 households) from each village or community was selected, and from those households, 1 permanent resident aged 18 years or older was selected by the Kish grid method (8,9). Permanent residents were defined as those who had lived in the surveillance counties or districts for at least 6 months. Short-term residents and migrants (ie, those living less than 6 months at that residence) were excluded from the survey. Thus, these data are nationally representative of permanent residents.

A face-to-face interview using standardized questionnaires was conducted by trained investigators. We made at least 3 attempts to contact the selected resident. A household with similar structure (eg, family size and socioeconomic status in the same village or community neighborhood) was used as replacement if we failed to reach the selected household resident. The interviews were administered at a convenient and accessible site or at the residents’ homes.

A total of 98,712 people were enrolled and interviewed from August to December of 2010. All nonrespondents were replaced, and the overall sample replacement rate (nonresponse rate) was 9.3%. For this analysis we examined data from female participants aged 18 years or older. The survey received ethics approval from the Ethics Committee of the Chinese Center for Disease Control and Prevention. Informed consent was obtained from all study participants.

### Cancer screening and measurements

Breast cancer screening participation and its frequency were determined by the following 2 questions: “Have you ever had a breast cancer examination?” (Answers: yes, no, don’t know). A positive response was interpreted as having participated in any method of screening (ie, mammogram, ultrasound, or clinical breast examination). Those who answered yes were further queried: “If you ever have had, how many years ago was your last examination?”

For our analyses, we used 6 age groups (<30, 30–39, 40–49, 50–59, 60–69,and ≥70), 2 ethnic groups (Han and other [includes Zhuang, Manchu, Hui, Miao, Uyghur, Yi, Tujia, Mongol, Korean, and Tibetan]), 3 education levels (<6 years, 6–11 years, and ≥12 years of school education), 3 types of marital status (never married, married, and other [includes those living with partner, widowed, divorced, and married but not living with husband]), 3 occupational categories (employed, student or unemployed, and retired), and 3 medical insurance categories (urban insurance, rural insurance, and no insurance). Urban insurance included basic medical insurance for urban employees, government payment for government employees, commercial insurance, supplementary insurance for urban residents, and insurance for serious diseases. Rural insurance included coverage from the New Rural Cooperative Medical System. Provinces, autonomous regions, and municipalities were classified into east (n = 8), central (n = 11) and west (n = 12) areas according to the China National Bureau of Statistics. Each geographic area included both urban and rural areas.

### Statistical analysis

We used SAS (version 9.2; SAS Institute, Cary, North Carolina) to account for the complex sampling design for these analyses. Estimates were adjusted to the national population consistent with the CCDRFSS complex sampling design and accounted for stratification, primary sampling units, and clustering. We weighted all calculations to obtain DSP representative results consistent with the sampling scheme. Poststratification adjustments for age and sex using data from the 2009 National Sample Survey on Population Changes were also made. Taylor’s series methods were used, including finite population correction to estimate standard errors.

We used descriptive statistics to examine demographic characteristics and breast cancer screening participation rates. We calculated prevalence ratios by comparing screened and unscreened participants across sociodemographic groups. To further explore determinants that influenced breast cancer screening, we applied logistic regression to examine the relationship between sociodemographic status and breast cancer screening, and we calculated odds ratios (ORs). We calculated 95% confidence intervals (CIs) for our estimates and used a *P* value of less than .05 to define significance.

## Results

A total of 53,513 women aged 18 to 107 years participated (mean age, 45 years; standard deviation [SD] = 11); approximately 66% of women were younger than 50 years, and approximately 40% were from the eastern region. (Table 1)

**Table 1:** Characteristics of Female Participants (N = 53,513), China Chronic Disease and Risk Factor Surveillance System, 2010

Characteristic	n (%)^a^
**Age, y**	
<30	7,470 (24.8)
30–39	9,953 (20.4)
40–49	14,003 (21.2)
50–59	11,626 (16.8)
60–69	6,721 (9.4)
≥70	3,740 (7.3)
**Region**	
Eastern	18,096 (40.3)
Middle	16,536 (32.4)
Western	18,881 (27.3)
**Residence**	
Urban	21,647 (31.4)
Rural	31,866 (68.6)
**Ethnicity**	
Han	45,523 (90.5)
Other^b^	7,990 (9.5)
**Education, y**	
<6	16,538 (26.6)
6-11	25,271 (50.3)
≥12	11,704 (23.1)
**Marital status**	
Never married	3,350 (10.6)
Married	45,002 (81.2)
Other^c^	5,161 (8.2)
**Occupation**	
Employed	35,686 (69.2)
Student/unemployed	13,032 (24.6)
Retired	4,795 (6.2)
**Medical insurance^d^**	
Urban	16,431 (26.6)
Rural	34,661 (68.3)
None	2,421 (5.0)

a Percentages were weighted to represent the total population of the national disease surveillance points system with poststratification for age and sex.

b Other ethnicity includes Zhuang, Manchu, Hui, Miao, Uyghur, Yi, Tujia, Mongol, Korean, and Tibetan.

c Other marital status includes those living with partner, widowed, divorced, and married but not living with husband.

d Urban insurance included basic medical insurance for urban employees, government payment for government employees, commercial insurance, supplementary insurance for urban residents, and insurance for serious diseases. Rural insurance included the New Rural Cooperative Medical Scheme, which is the primary form of medical security provided to Chinese farmers at present.

Overall, 21.7% (95% CI, 19.2%–24.2%) of women aged 18 years or older reported ever being screened for breast cancer (Table 2). Women 60 years or older had the lowest breast cancer screening participation. Higher rates were found among the intermediate age groups; 30.7% (95% CI, 26.9%–34.4%) of women aged 30 to 39 years and 30.5% (95% CI, 26.7%–34.2%) of women aged 40 to 49 years were screened. Compared with women aged 70 years or older, those younger than 30 were 2.2 times as likely (95% CI, 1.8–2.7, *P* < .001), those aged 30 to 39 were 4.8 times as likely (95% CI, 4.0–5.8, *P* < .001), those aged 40 to 49 were 4.8 times as likely (95% CI, 4.0–5.7, *P* < .001), those aged 50 to 59 were 3.7 times as likely (95% CI, 3.1–4.3, *P* < .001), and those aged 60 to 69 were 1.8 times as likely (95% CI, 1.5–2.1, *P* < .001) to be screened (Table 2).

**Table 2:** Prevalence of Breast Cancer Screening and Its Associations With Demographic and Socioeconomic Characteristics, China Chronic Disease and Risk Factor Surveillance System, 2010

Group	Subjects	Ever Screened	% (95% CI)^a^	Prevalence Ratio (95% CI)	Unadjusted OR (95% CI)	Adjusted OR^b^ (95% CI)
**Total**	52,382	12,138	21.7 (19.2–24.2)	NA	NA	NA
**Demographics**						
**Age, y**						
<30	7,393	1,063	13.7 (11.9–15.5)	2.2 (1.8–2.7)^c^	2.3 (1.9–2.9)^c^	2.8 (1.9–4.3)^c^
30–39	9,813	3,014	30.7 (26.9–34.4)	4.8 (4.0–5.8)^c^	6.5 (5.2–8.1)^c^	2.3 (1.6–3.3)^c^
40–49	13,788	4,326	30.5 (26.7–34.2)	4.8 (4.0–5.7)^c^	6.5 (5.3–7.9)^c^	2.1 (1.6–3.0)^c^
50–59	11,402	2,629	23.3 (19.9–26.7)	3.7 (3.1–4.3)^c^	4.5 (3.8–5.3)^c^	1.7 (1.2–2.4)^c^
60–69	6,528	844	11.2 (9.4–13.1)	1.8 (1.5–2.1)^c^	1.9 (1.5–2.3)^c^	1.4 (1.0–2.1)^c^
≥70	3,458	262	6.3 (5.1–7.6)	1 [Reference]		
**Region**						
Eastern	17,658	5,226	27.4 (22.5–32.3)	1.7 (1.3–2.1)^c^	1.9 (1.4–2.6)^c^	1.5 (1.2–2.0)^d^
Middle	16,300	3,647	18.8 (14.6–23.1)	1.1 (0.9–1.5)	1.2 (0.8–1.6)	1.0 (0.9–1.3)
Western	18,424	3,265	16.5 (14.2–18.9)	1 [Reference]		
**Residence**						
Urban	21,233	7,239	34.1 (30.2–37.9)	2.1 (1.7–2.7)^c^	2.7 (2.1–3.6)^c^	0.9 (0.7–1.1)
Rural	31,149	4,899	16.0 (13.0–18.9)	1 [Reference]		
**Ethnicity**						
Han	44,630	11,171	22.4 (19.7–25.0)	1.5 (1.1–2.0)^d^	1.6 (1.2–2.3)^d^	1.0 (0.7–1.4)
Other^e^	7,752	967	14.8 (10.7–18.8)	1 [Reference]		
**Socioeconomics**						
**Education, y**						
<6	15,900	1,398	9.0 (7.3–10.8)	1 [Reference]		
6-11	24,885	5,834	21.2 (18.7–23.8)	2.3 (2.1–2.7)^c^	2.7 (2.4–3.1)^c^	1.0 (0.9–1.3)^c^
≥12	11,597	4,906	36.6 (33.0–40.2)	4 (3.4–4.9)^c^	5.8 (4.7–7.2)^c^	1.2 (1.0–1.6)^c^
**Marital status**						
Never married	3,315	268	7.3 (5.6–8.9)	1 [Reference]		
Married	44,153	11,122	24.3 (21.4–27.2)	3.3 (2.7–4.2)^c^	4.1 (3.2–5.3)^c^	0.7 (0.4–1.1)^c^
Other^f^	4,914	748	14.0 (11.8–16.2)	1.9 (1.5–2.4)^c^	2.1 (1.6–2.7)^c^	0.6 (0.3–0.9)^c^
**Occupation**						
Employed	35,023	8,062	21.8 (19.2–24.4)	0.6 (0.5–0.7)^c^	0.5 (0.4–0.6)^c^	1.6 (1.2–2.1)^c^
Student/ unemployed	12,694	2,389	17.2 (14.2–20.1)	0.5 (0.4–0.5)^c^	0.3 (0.3–0.4)^c^	1.3 (1.0–1.8)^g^
Retired	4,665	1,687	38.0 (33.1–42.9)	1 [Reference]		
**Medical insurance^h^**						
Urban	16,121	6,554	40.6 (37.1–44.0)	2.6 (2.3–3.0)^c^	3.7 (3.1–4.4)^c^	1.3 (1.0–1.7)^g^
Rural	33,877	5,158	14.7 (12.4–17.0)	0.9 (0.8–1.1)	0.9 (0.7–1.2)	1.0 (0.7–1.3)
None	2,384	426	15.6 (13.1–18.1)	1 [Reference]		

Abbreviations: CI, confidence interval; OR, odds ratio; NA, not applicable.

a Percentages were weighted to represent the total population of the national disease surveillance points system with poststratification for age and sex.

b All demographic and socioeconomic factors identified in the table were included in the logistic analysis model.

c *P* < .001.

d *P* < .01 and ≥ .001.

e Other ethnicity includes Zhuang, Manchu, Hui, Miao, Uyghur, Yi, Tujia, Mongol, Korean, and Tibetan.

f Other marital status includes those living with partner, widowed, divorced, and married but not living with husband.

g *P* < .05 and ≥ .01.

h Urban insurance included basic medical insurance for urban employees, government payment for government employees, commercial insurance, supplementary insurance for urban residents, and insurance for serious diseases. Rural insurance included the New Rural Cooperative Medical Scheme, which is the primary form of medical security provided to Chinese farmers at present.

The screening prevalence was highest in the east (27.4%, 95% CI, 22.5%–32.3%), followed by the middle area (18.8%, 95% CI, 14.6%–23.1%) and lowest in the west (16.5%, 95% CI, 14.2%–18.9%) (Table 2). The unadjusted breast cancer screening rate in the eastern region was 1.5 times that of the western region (95% CI, 1.2–2.0; *P* < .01); no significant difference was found between the middle and western regions. The proportion of urban women who had ever received breast cancer screening (34.1%; 95% CI, 30.2%–37.9%) was more than twice than that among rural women (16.0%; 95% CI, 13.0%–18.9%). However, after adjusting for other factors, this difference was not significant (*P* = .67).

The prevalence of breast cancer screening increased with increasing education levels (Table 2) ranging from 9.0% (95% CI, 7.3%–10.8%) among women with 6 or less years of education to 36.6% (95% CI, 33.0%–40.2%) among those with more than 12 years of education. Married women had the highest level of breast cancer screening (24.3%; 95% CI, 21.4%–27.2%), while those who had never married had the lowest level (7.3%; 95% CI, 5.6%–8.9%, *P* < .001). Overall, 40.6% (95% CI, 37.1%–44.0%) of participants with urban insurance were screened while lower levels were found among those with rural insurance (14.7%; 95% CI, 12.4%–17.0%) and no insurance (15.6%; 95% CI, 13.1%–18.1%). However, when adjusting for other factors, compared with those with no insurance the likelihood of screening among those with urban (adjusted OR = 1.3; 95% CI, 1.0–1.7) and rural (adjusted OR = 1.0; 95% CI, 0.7–1.3) insurance was not significantly different.

Of the 12,138 women who had ever been screened for breast cancer, 11,956 reported the time of their last screening. Among those who had been screened, 72.5% were screened within 2 years before the survey (Figure). The proportion screened in the last 2 years was higher for age groups younger than 30, 30 to 39, 40 to 49, 50 to 59, and 60 to 69 years; using our logistic regression model we found these age groups to be 2.9, 2.3, 2.2, 1.7, and 1.4 times as likely as those aged 70 or older to be screened within the last 2 years. Furthermore, the proportion screened in the past 2 years tended to decline moving from the eastern to the middle then to the western region. Women with urban medical insurance were 1.3 (95% CI, 1.0–1.7; *P* = .04) times as likely to report screening within the past 2 years as those without insurance (Table 3).

**Figure:**
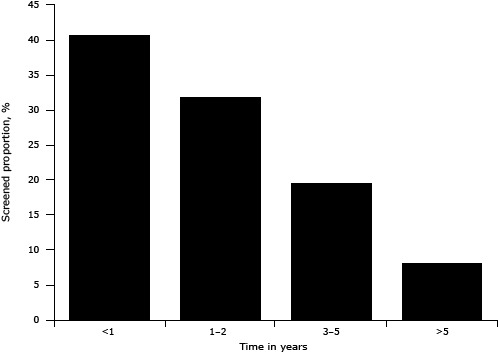
Distribution of duration since last breast cancer screening, China Chronic Disease and Risk Factor Surveillance System, 2010. Percentages were weighted to represent the total population of the national disease surveillance points system with poststratification for age and sex. **Table:** 

Time From Last Breast Cancer Screening, Years	No. Screened (%)
<1	4,759 (40.6)
1–2	3,718 (31.9)
3–5	2,342 (19.4)
>5	1,137 (8.1)
Total	11,956 (100)

**Table 3:** Association Between Characteristics of Participants and Breast Cancer Screening Within the Past Two Years, China Chronic Disease and Risk Factor Surveillance System, 2010

Group	No. Screened	No. Screened ≤2 Years (%)	OR (95% CI)^a^	*P* Value
**Total**	11,956	8,477 (72.5)	NC	
**Age, y**				
<30	1,039	820 (80.1)	2.9 (1.9–4.4)	<.001
30–39	2,965	2,246 (74.8)	2.3 (1.6–3.3)	<.001
40–49	4,263	3,126 (73.5)	2.2 (1.6–3.0)	<.001
50–59	2,597	1,685 (66.6)	1.7 (1.2–2.4)	.001
60–69	833	471 (60.0)	1.4 (1.0–2.1)	.06
≥70	259	129 (49.3)	1 [Reference]	
**Region**				
Eastern	5,124	3,823 (75.9)	1.5 (1.2–2.0)	.001
Middle	3,629	2,453 (69.2)	1.0 (0.9–1.3)	.64
Western	3,203	2,201 (68.7)	1 [Reference]	
**Residence**				
Urban	7,185	5,022 (72.0)	1.0 (0.8–1.2)	.67
Rural	4,771	3,455 (73.0)	1 [Reference]	
**Ethnicity**				
Han	11,012	7,804 (72.5)	1.0 (0.7–1.4)	.91
Other^b^	944	673 (72.9)	1 [Reference]	
**Education, y**				
<6	1,364	876 (64.9)	1 [Reference]	
6–11	5,727	3,986 (70.8)	1.0 (0.8–1.2)	.79
≥12	4,865	3,615 (76.7)	1.2 (0.9–1.6)	.16
**Marital status**				
Never married	265	222 (86.0)	1 [Reference]	
Married	10,949	7,796 (72.5)	0.7 (0.4–1.1)	.11
Other^c^	742	459 (63.4)	0.6 (0.3–0.9)	.02
**Occupation**				
Employed	7,943	5,871 (75.1)	1.6 (1.2–2.2)	.001
Student/unemployed	2,339	1,609 (69.4)	1.4 (1.0–1.8)	.03
Retired	1,674	997 (61.3)	1 [Reference]	
**Medical insurance^d^**				
Urban	6,504	4,661 (74.6)	1.3 (1.0–1.7)	.04
Rural	5,028	3,524 (70.2)	1.0 (0.8–1.4)	.93
None	424	292 (71.6)	1 [Reference]	

Abbreviations: OR, odds ratio; CI, confidence interval; NC, not calculated.

a Age group, region, education, marital status, occupation, and medical insurance identified in the table were included in the logistic analysis model.

b Other ethnicity includes Zhuang, Manchu, Hui, Miao, Uyghur, Yi, Tujia, Mongol, Korean, and Tibetan.

c Other marital status includes those living with partner, widowed, divorced, and married but not living with husband.

d Urban insurance included basic medical insurance for urban employees, government payment for government employees, commercial insurance, supplementary insurance for urban residents, and insurance for serious diseases. Rural insurance included the New Rural Cooperative Medical Scheme, which is the primary form of medical security provided to Chinese farmers at present.

## Discussion

Breast cancer is the most commonly diagnosed cancer in China and it may become more common in the future considering past trends and the ongoing rapid transition toward diets and lifestyles that may increase risk (10,11). We found that the overall breast cancer screening participation rate among Chinese women was only 21.7%.

WHO indicates that the desired outcome objective (within 5 years) of launching a breast cancer screening program is to achieve more than 70% coverage of women older than 50 years with mammography screening every 2 years (4). We found participation rates far lower than the WHO desired goal, as well as lower than those found in other countries. For example, the rate of breast cancer screening among Chinese women is lower than that among US women. Based on data from the 2010 United States National Health Interview Survey, 72.4% of women aged 50 to 74 years were screened for breast cancer by mammography during the past 2 years (12). In 2010 in England, the National Health Service Breast Screening Programme reported that 76.9% of women aged 53 to 70 were screened for breast cancer during the past 3 years (13). The screening rates among women 50 years or older in these countries was far higher than the rate among Chinese women. However, because of differences in methods and the study populations, direct comparisons should be made with caution. In China, we found the highest rates of breast cancer screening were among women aged 30 to 39 and 40 to 49 years and that screening participation decreased with aging, with the largest proportion of those never screened among women aged 50 years or older. WHO and the United States Preventive Services Task Force (USPSTF) (4,14) recognize that advanced age is a well-established risk factor for breast cancer, and increasing breast cancer screening among older women should be a priority, especially for those aged 50 to 69 years. Our study found that the proportion screened within the last 2 years among older women was lower than that among younger women, suggesting that targeted screening uptake efforts among elder women should be a priority.

We found a significant difference in breast cancer screening participation between women from eastern and western China. There are vast differences in sociocultural and economic development and health-care access across China. Eastern China is generally more developed than middle China, and western China is the least developed, which may explain the higher screening rates in the east. Economic levels depend more on geographic region than on rural versus urban areas, as many urban cities in western China are less developed than the rural villages in eastern China. These may be the reason for our finding that the adjusted OR of rural versus urban women being screened was not significant, while the unadjusted OR was significant. Consistent with previous studies (15,16), we found that screening participation increased with education level, suggesting that awareness of breast cancer screening should be increased among women with lower education levels to improve participation rates. In China, socioeconomic indicators predict health awareness, health care access, and affordability through economic, psychological, and social mediators (17,18). We also found that women with urban insurance tended to be more likely to undergo breast cancer screening than those with rural insurance or without insurance. In China, the shift to a market economy was accompanied by the demise of the rural communes in the 1980s, and the rural health insurance system based on the rural communes became devoid (19). The subsequent New Rural Cooperative Medical Scheme (NRCMS), which has a low reimbursement level for outpatient services of rural residents, was not initiated until 2002 (20). In general, compared with the urban insurance system, NRCMS does not have standard reimbursement policies across different areas, and coverage rates for clinical preventive services and medicines are lower (20–22).

Cancer screening programs should be evidence-based, and recommendations should be focused on those interventions for which there is sufficient evidence of efficacy and cost-effectiveness. In low- and middle-income countries, including China, organized mammography screening is neither affordable nor feasible, leaving clinical breast examination as an alternative screening option (23). Although its effectiveness in reducing breast cancer deaths is not known, clinical breast examination may be of particular importance in countries where there are insufficient resources and where most tumors diagnosed are in an advanced stage (4,24).

In China, to increase participation, free breast cancer examinations have been offered by governments in some rural districts. For example, the Free Examination For “Two Cancers” Programme Among Rural Women, jointly promoted by the China Ministry of Health and the China Women’s Federation, provides breast and cervical cancer screening for poor rural women. For the breast cancer screening component, clinical breast examination, mammography, and ultrasound examination are all covered (25). From 2009 through 2011, 1.46 million rural women were screened for breast cancer by 1 or 2 of these methods. WHO indicates that breast cancer screening by mammography alone in countries that can feasibly implement mammography, with follow-up of individuals who have confirmed diagnoses or results indicating the likelihood of cancer, would reduce mortality from breast cancer by up to one-third among women aged 50 to 69 years (3,26). The USPSTF recommends biennial screening for women aged 50 to 74 years using mammography (14). There is limited evidence for its effectiveness for women 40 to 49 years of age (27,28). The evidence base for using physical examination of the breasts as a single screening modality is inadequate, although there are indications that good clinical examinations by specially trained health workers could play an important role in the areas with limited resources (27). In India, one study found that 3 rounds of triennial clinical breast examination reduced the incidence rate of advanced disease and breast cancer mortality (23). In the Philippines, a randomized trial of screening for breast cancer by clinical examination found that the survival of women who were screened and diagnosed with breast cancer was significantly better than that of women who were not screened (29).

Finally, implementation of breast cancer screening programs must consider local area social and economic realities. As a developing country, China must give priority to support rural women and those from the western area and find low-cost screening strategies. In view of competing priorities for many preventive care efforts and budget limitations in China, scaling up to full national coverage represents a much greater challenge than that for more developed countries.

Our study has many strengths. First, it is the largest study of breast cancer screening in China. Second, we included a nationally representative sample selected from all 31 provinces, autonomous regions, and municipalities in China. Third, we achieved a high response rate, and our data were recently collected and should prove useful for understanding the current situation and for strategic programmatic cancer planning. Fourth, we assessed the independent associations of the key risk factors, through which effects of interrelated determinants such as age, education, and employment can be better understood.

Our study also has limitations. First, we lacked information on the type of screening method. Our query did not ask for the method of screening, and as such, it includes clinical breast examination, mammography, and other possible examinations. As our study is based on the national surveillance focusing on chronic diseases, and risk factors and mammography screening is neither affordable nor feasible in most of areas in China, we did not collect detailed information on the type of screening, making it difficult to compare our findings with participation rates for mammography screening behaviors in other countries. A second limitation is that breast cancer screening participation was self-reported, which might be associated with an information bias.

The key public health challenges for breast cancer screening are first to initiate screening and second to maintain screening behaviors. Regional area, education level, and medical insurance status significantly influenced uptake of breast cancer screening. Tailor-made strategic promotion programs targeting older women with low educational levels may enhance awareness and acceptance within this vulnerable, high-risk group. Successful strategies to increase participation in breast cancer screening include community programs with invitation letters, mailed educational materials, invitation letters with follow-up telephone calls, and direct reminders for the women (30). Promotion of breast cancer screening will need policies that address Chinese women from the west, from rural areas, with low education levels, and without medical insurance.
